# Resuming Training in High-Level Athletes After Mild COVID-19 Infection: A Multicenter Prospective Study (ASCCOVID-19)

**DOI:** 10.1186/s40798-022-00469-0

**Published:** 2022-06-25

**Authors:** Laurent Chevalier, Hubert Cochet, Saagar Mahida, Sylvain Blanchard S, Antoine Benard, Tanguy Cariou, Soumaya Sridi-Cheniti, Samy Benhenda, Stéphane Doutreleau, Stéphane Cade, Sylvain Guerard, Jean-Michel Guy, Pascale Trimoulet, Stéphane Picard, Bernard Dusfour, Aurelie Pouzet, Stéphanie Roseng, Marco Franchi, Pierre Jaïs, Isabelle Pellegrin, ASCCOVID Investigators, Laurent Chevalier, Isabelle Pellegrin, Michel Babeau, Akram Bensaïd, Jeremy Bernard, Sylvain Blanchard, Cédric Chadourne, Patrick Claisse, Victor Cubillé, Jean-Michel Dindart, Marco Franchi, Sophie Goguillot, Ludovic Humetz, Philippe Izard, Benjamin Laffourcade, Bernard Lemahieu, Damien Monnot, Cédric Poirier, Philippe Pouget, Jean-Louis Rebeyrol, Elliot Rubio, Carlos Vela, Quentin Vincent

**Affiliations:** 1 Clinique du Sport Bordeaux-Mérignac, 2 rue Georges Negrevergne, 33700 Merignac, France; 2 IHU Liryc, CHU Bordeaux, Univ. Bordeaux, Bordeaux, France; 3 Liverpool Heart and Chest Hospital NHS Trust, Liverpool, UK; 4 IHU LIRYC, Bordeaux, France; 5 French National Rugby League Medical Committee, Montpellier, France; 6 Public Health Department, Clinical Epidemiology Unit, CHU Bordeaux, 33000 Bordeaux, France; 7 Department of Cardio-Vascular Imaging, CHU Bordeaux, Univ. Bordeaux, 33000 Bordeaux, France; 8 HP2 Laboratory, Université Grenoble Alpes, INSERM, CHU Grenoble Alpes, Grenoble, France; 9 Clinique du Millénaire, Montpellier, France; 10 Department of Cardiology, Desgenettes Hospital, Lyon, France; 11 Cardiorespiratory Rehabilitation Centre Clos Champirol, Saint-Priest-en-Jarez, France; 12 Laboratory of Virology, CHU Bordeaux, 33000 Bordeaux, France; 13 Department of Biology, CHU Bordeaux, 33000 Bordeaux, France; 14 Translational Research Project Manager, Department of Biology, CHU Bordeaux, 33000 Bordeaux, France; 15 SMART Campus Olympique, Université Bordeaux, Bordeaux, France; 16 Department of Electrophysiology and Cardiac Stimulation, CHU Bordeaux, 33000 Bordeaux, France; 17 Univ. Bordeaux, Bordeaux, France; 18 Laboratory of Immunology and Immunogenetics, CHU Bordeaux, 33000 Bordeaux, France; 19 CNRS, UMR 5164-ImmunoConcEpT, Bordeaux University, Bordeaux, France

**Keywords:** Sport, SARS-CoV-2, ECG, Echocardiography, Stress test, Cardiac MRI

## Abstract

**Background:**

There is a paucity of data on cardiovascular sequelae of asymptomatic/mildly symptomatic SARS-Cov-2 infections (COVID).

**Objectives:**

The aim of this prospective study was to characterize the cardiovascular sequelae of asymptomatic/mildly symptomatic COVID-19 among high/elite-level athletes.

**Methods:**

950 athletes (779 professional French National Rugby League (F-NRL) players; 171 student athletes) were included. SARS-Cov-2 testing was performed at inclusion, and F-NRL athletes were intensely followed-up for incident COVID-19. Athletes underwent ECG and biomarker profiling (D-Dimer, troponin, C-reactive protein). COVID(+) athletes underwent additional exercise testing, echocardiography and cardiac magnetic resonance imaging (CMR).

**Results:**

285/950 athletes (30.0%) had mild/asymptomatic COVID-19 [79 (8.3%) at inclusion (COVID(+)_prevalent_); 206 (28.3%) during follow-up (COVID(+)_incident_)]. 2.6% COVID(+) athletes had abnormal ECGs, while 0.4% had an abnormal echocardiogram. During stress testing (following 7-day rest), COVID(+) athletes had a functional capacity of 12.8 ± 2.7 METS with only stress-induced premature ventricular ectopy in 10 (4.3%). Prevalence of CMR scar was comparable between COVID(+) athletes and controls [COVID(+) vs. COVID(−); 1/102 (1.0%) vs 1/28 (3.6%)]. During 289 ± 56 days follow-up, one athlete had ventricular tachycardia, with no obvious link with a SARS-CoV-2 infection. The proportion with troponin I and CRP values above the upper-limit threshold was comparable between pre- and post-infection (5.9% vs 5.9%, and 5.6% vs 8.7%, respectively). The proportion with D-Dimer values above the upper-limit threshold increased when comparing pre- and post-infection (7.9% vs 17.3%, *P* = 0.01).

**Conclusion:**

The absence of cardiac sequelae in pauci/asymptomatic COVID(+) athletes is reassuring and argues against the need for systematic cardiac assessment prior to resumption of training (clinicaltrials.gov; NCT04936503).

**Supplementary Information:**

The online version contains supplementary material available at 10.1186/s40798-022-00469-0.

## Key points

• The rate of adverse clinical cardiac events after a mild/asymptomatic COVID-19 infection is very low, despite resumption of high-level training
• In the vast majority of athletes with a mild/asymptomatic COVID-19 infection, biomarkers of cardiac injury remain in the normal range and the incidence of structural cardiac abnormalities is very low
• Among the small subset of athletes with structural/electrical abnormalities potentially related to mild COVID-19, the abnormalities are subtle and restricted to a single cardiac investigation.

## Introduction

Cardiovascular complications among hospitalized patients with severe COVID-19 have been reported in 15 to 20% of cases. Potential complications include myocardial injury secondary to myocarditis, arrhythmias and thromboembolic events [1–6]. Cardiovascular Magnetic Resonance (CMR) imaging in these patient has been associated with a range of abnormalities, both during the acute [7–9] or convalescent [10] phase. The prevalence of CMR abnormalities is highly variable, with significant heterogeneity between patients. There is currently a relative paucity of data on cardiovascular sequelae among patients with asymptomatic or mildly symptomatic COVID-19 [11].

Athletes who undertake a high-level of training represent a particularly valuable cohort to understand the cardiovascular impact of milder SARS-CoV2 infections. Indeed, competitive athletes represent a unique population that may be at high risk for situational transmission of disease, and once infected, may be at risk for sudden cardiac death (SCD) during competitive training [12–14]. Asymptomatic viral myocarditis is a common cause of SCD, especially among young patients [15, 16]. The reported prevalence of CMR abnormalities in asymptomatic/mildly symptomatic athletes after SARS-CoV2 infection is also highly heterogeneous (0%-15%) [17–23]. Of note, some of these CMR studies did not involve imaging in control subjects. In this context, understanding the impact of COVID-19 has important implications, both in terms of risk stratification and development of guidelines to support the resumption of training.

The aim of the present study was to characterize the cardiovascular sequelae of asymptomatic or mildly symptomatic COVID-19 infections among high-level/elite athletes, and by implication, the potential role of systematic cardiovascular assessment in these athletes. We performed a prospective comprehensive cardiovascular evaluation using ECG, exercise stress testing, echocardiography, high-resolution cardiac magnetic resonance imaging (HR-CMR with case and control subjects, and blindly interpreted images) and pre- and post-COVID-19 biomarker profiling (D-Dimer, troponin, C-reactive protein [CRP]).

## Material and methods

### Study design and population

The ASCCOVID study (clinicaltrials.gov; NCT04936503; https://clinicaltrials.gov/ct2/results?cond=&term=NCT04936503&cntry=&state=&city=&dist=; date of registration; date of registration 23 June 2021) is a prospective, multicenter, nationwide cohort study which enrolled athletes from two distinct cohorts: professional athletes from the French National Rugby League (F-NRL, XV players rugby) and high-level sports students, whose sports profile is more representative of leisure sports in the general population. Written informed consent was obtained from all participants. The study was conducted in accordance with the Helsinki Declaration after the approval of the Ethics Committee (CPP Sud Ouest II, ID-RCB: 2020-A01196-33) and authorization of French Competent Authority (ANSM).

The F-NRL includes two French professional rugby male union divisions. F-NRL players were consecutively enrolled when training resumed in June 2020. The F-NRL training regime consisted of 20–25 h of intensive training per week. Following the F-NRL protocol, a complete pre-season checkup was scheduled for all players and corresponded to the day of enrollment (D0). Baseline assessment included a standardized questionnaire, clinical examination, ECG and blood sampling (CRP, troponin I, D-Dimer, SARS-CoV2 serology). Players subsequently underwent daily monitoring for symptoms of SARS-COV2 infection and twice-weekly nasopharyngeal RT-PCR. Players were considered COVID(+) if they had: (1) positive SARS-CoV2 RT-PCR in the previous 3 months and/or positive SARS-CoV2 serology at inclusion (COVID(+)_prevalent_) or (2) positive SARS-CoV2 RT-PCR during season follow-up (COVID(+)_incident_). COVID(+)_incident_ players with positive SARS-CoV2 RT-PCR during the season were subject to 7-days enforced rest and isolation. The rest period was based on the F-NRL protocol. Players stopped all physical activity for 7 days, and on day 8, they resumed physical activity in isolation. ECG, echocardiography and stress testing were performed between day 10 and day 14. Resumption of full training on day 15 was dependent on normal cardiological assessment.

In parallel, students athletes of the University of Bordeaux, with lower exercise intensity (8–12 h/week), were enrolled at the start of the academic year (October 2020). The student athlete cohort was assessed with a standardized questionnaire, ECG and SARS-CoV2 serology. Student athletes were considered COVID(+) if they had (1) positive SARS-CoV2 PCR in the previous 6 months and/or (2) positive serology at inclusion (COVID(+)_prevalent_). COVID(+) student athletes underwent baseline ECG, echocardiography and stress testing. Resumption of sport was conditional on the absence of abnormalities on cardiac assessment. They did not receive specific clinical or biological COVID follow-up visit during the academic year. Clinical status was reviewed at the end of the academic year (eight months post-inclusion).

### Cardiac investigations

A 12-lead ECG was performed at inclusion in all F-NRL athletes and compared to an ECG from the previous year. Various models of CE marked 12-lead ECG were used with standard parameters (1 mV = 10 mm and 25 mm/s) and 0.05–100 Hz. For COVID(+)_incident_ F-NRL players, the post-COVID ECG was compared to pre-COVID ECG. COVID(+) players (prevalent or incident) underwent echocardiography (with analysis of biplane LV ejection fraction, segmental wall motion, diastolic function and pericardial effusion). Stress testing was performed with monitoring for exercise-induced arrhythmias. Student athletes of the University of Bordeaux also underwent a baseline ECG at inclusion, and any COVID(+)_prevalent_ athletes (history of positive SARS-CoV-2 PCR in the previous 6 months and/or positive serology at inclusion) underwent echocardiography and stress tests.

Anonymized ECGs, echocardiograms, stress tests were analyzed in a core laboratory by six cardiologists with expertise in sports cardiology. Secondary reporting was performed by one of the six aforementioned experts. In cases of disagreement, the data were reviewed by all six cardiologists to reach consensus. Cardiac MRI was offered to all enrolled F-NRL players and student athletes. SARS-CoV-2 non-infected players and athletes [COVID(−)] were selected for MRI by using random draws without replacement, stratified by gender, in order to keep the COVID(+) and COVID(−) populations comparable with respect to their sex ratio.

MRI scanning was not performed in the first 14 days after infection, but rather from the 2nd month following the diagnosis of COVID-19 (positive PCR in rugby players and in relation to the onset of symptoms in student athletes who were only diagnosed with a lag on serologies). This approach ensured that any evolving MRI abnormalities were detected. A detailed MRI protocol is included in Additional file 1 section. Briefly, anonymized images were analyzed in a core laboratory at the Bordeaux University Hospital for left ventricular wall dimensions, left and right ventricular volumes, ejection fraction, global longitudinal strain, regional wall motion and pericardial effusion. Analysis of LGE was performed to detect myocardial or pericardial injury. T1 and T2 images were analyzed to measure mean T1/T2 values.

### Measurement of biomarkers

SARS-CoV-2 RT-PCR testing was conducted by nationally accredited laboratories. Serological samples were anonymized and centralized at Bordeaux University Hospital (IgG and IgM with ARCHITECT i2000SR immunoassay [Abbott, Illinois]). Throughout the study period, the D614G variant in the SARS-CoV-2 Spike was the predominant variant in France. At inclusion for student athletes and at three study time points for F-NRL players (inclusion, M1-M6, M9-M12 for COVID(+) players), biological samples were constituted and stored in the Center of Biological Resources (CRB-BBS, Bordeaux University Hospital). At inclusion, F-NRL rugby players’ biomarker levels (D-Dimer, troponin I and CRP) were measured. Biomarkers were collected at set intervals throughout the study.

### Statistical analyses

Sample size calculations were based on available estimates during the very early phase of the COVID-19 pandemic. Sample size was calculated based on an estimated 10% prevalence of COVID-19 seropositivity, and prevalence of arrhythmia of 2% in COVID(−) individuals versus 4% in COVID(+) individuals. Data are described as mean and standard deviation (SD), or median and interquartile range (IQR) for quantitative variables, and frequencies and percentages for qualitative variables. Comparison of cardiovascular and biological characteristics before and after COVID-19 used paired t tests for quantitative variables, and McNemar Chi-square tests for qualitative variables. Data analysis was performed using SAS software (Version 9.4).

## Results

### Study population

**Fig. 1:**
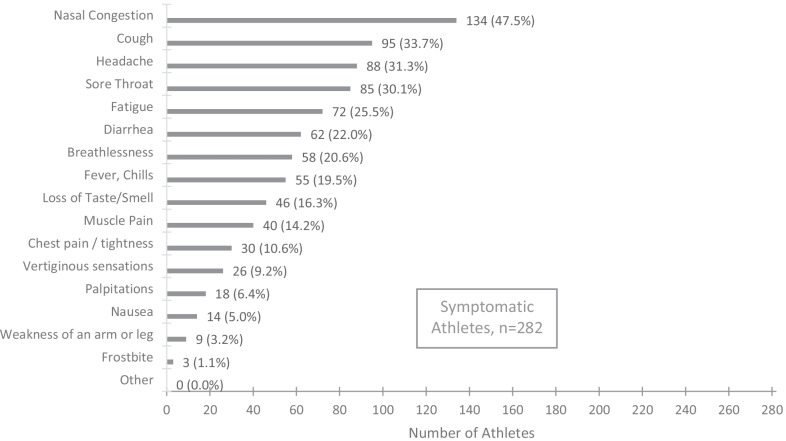
Reported symptoms evocative of SARS-CoV-2 infection in the study population (regardless of SARS-COV-2 test results). Athletes presenting only clinical signs of SARS-COV-2 infection (anosmia or ageusia) with negative serology and PCR were excluded from the analysis as we could not establish the diagnosis with a sufficient level of certainty

**Fig. 2:**
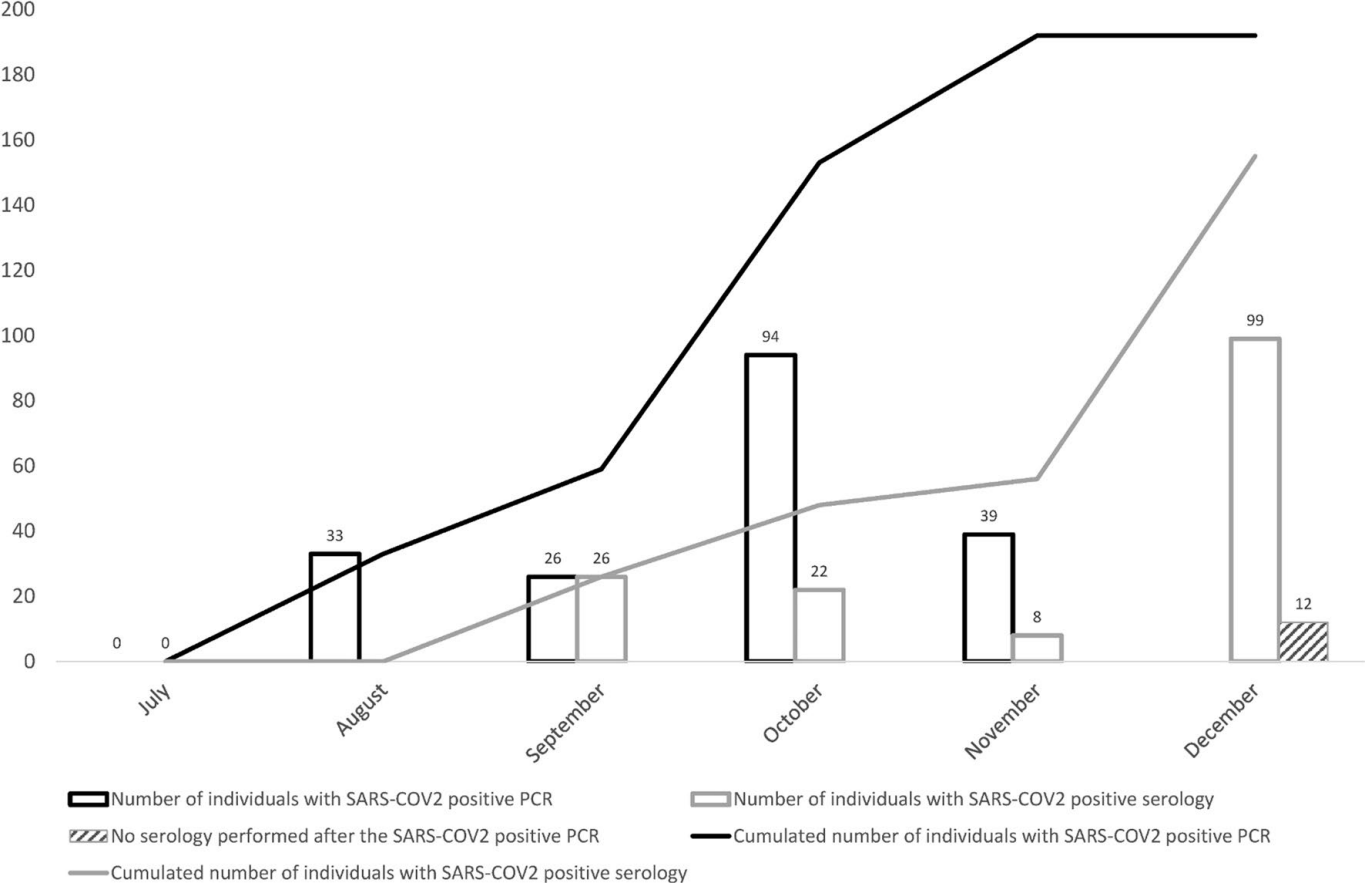
Occurrence of positive SARS-CoV-2 diagnosis during F-NRL season (RT-PCR and serologies). The F-NRL protocol involved twice-weekly SARS-CoV-2 RT-PCR testing (D614G variant). COVID(+) diagnosis was based on positive serology or a positive SARS-CoV-2 RT-PCR. 192/206 COVID(+)_incident_ cases were diagnosed by positive RT-PCR and 14 by positive serology (without previous positive RT-PCR). SARS-CoV-2 seroconversion (IgM and/or IgG) occurred in 155 (80.7%) of the 192 players diagnosed by RT-PCR, while 25 (13%) did not show detectable anti-SARS-CoV-2 antibodies during the follow-up period

**Fig. 3:**
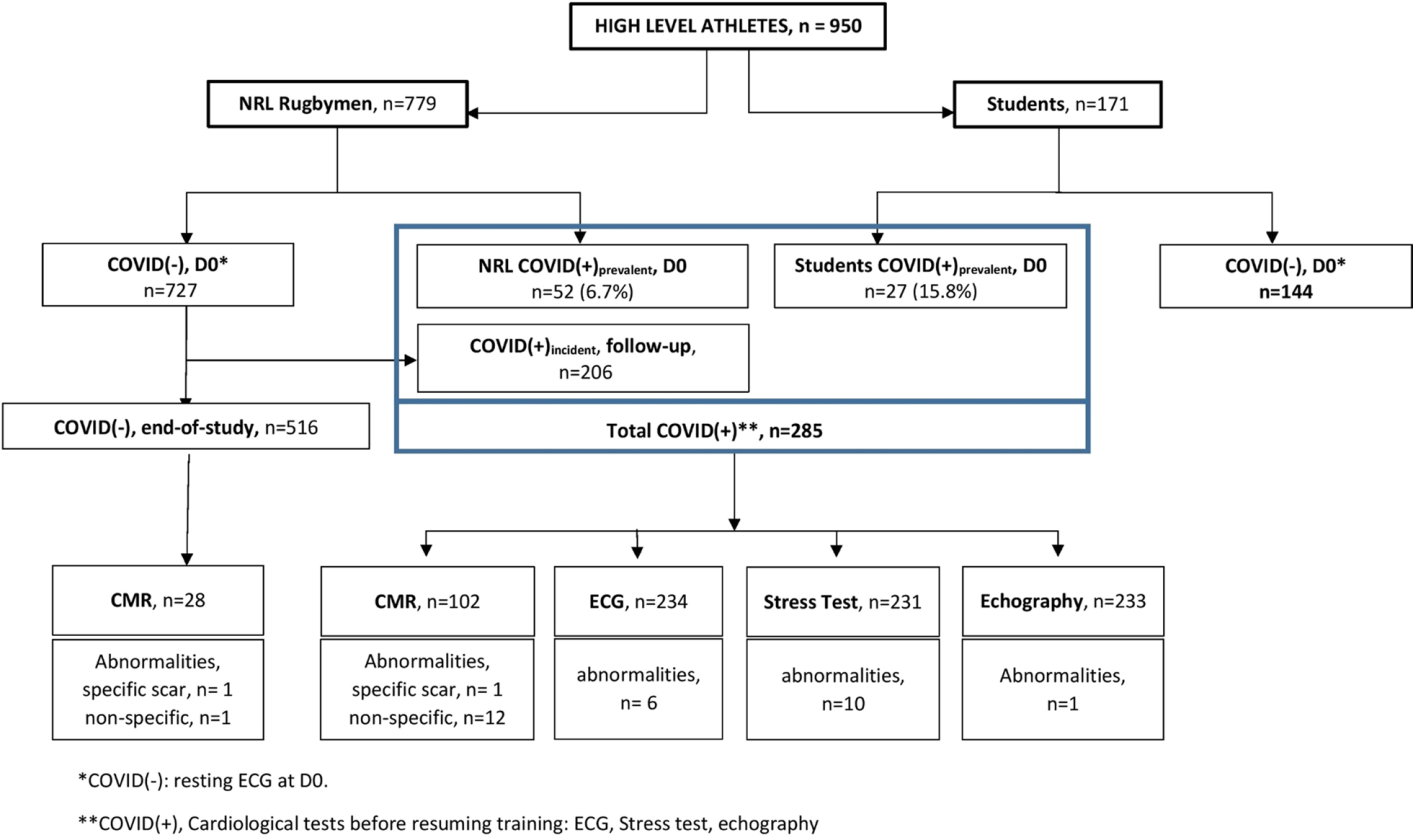
SARS-CoV-2 diagnosis and cardiovascular assessment results^1^ among athletes included in the ASCCOVID study

The ASCCOVID study took place between June 2020 and December 2020. The prospective cohort comprised of 779 F-NRL players (from 23 clubs in France), and 171 high-level sport students (from 27 sporting disciplines). Baseline characteristics are included in Table 1. 181/761 (24%) F-NRL players and 101/171 (59%) students reported at least one symptom of COVID-19 (Fig. 1). Among the 950 enrolled athletes, 79 (8%) were considered COVID(+) at inclusion [COVID(+)_prevalent_]. Only F-NRL players were monitored throughout the study period, enabling identification of incident [COVID(+)_incident_] cases. 206/727 (28%) COVID(−) F-NRL players at inclusion were diagnosed as COVID(+)_incident_, with RT-PCR or serology evidence, during the F-NRL season (Fig. 2). A flowchart of COVID-19 diagnosis and cardiovascular assessment is included in Fig. 3.

**Table 1:** Participant’s characteristics at inclusion and COVID-19 history

Characteristics	F-NLR Rugby players, *n* = 779	Student athletes^4^, *n* = 171
Sex, male, *n* (%)	779 (100.0)	85 (49.7)
Age (years), mean (SD)	25.8 (4.6)	20.1 (3.1)
BMI (Kg/m^2^), median (Q1; Q3)	29.0 (27.1; 31.9)	22.3 (20.7; 23.7)
BMI > 30, *n* (%)	310 (39.8)	5 (2.9)
*Ethnicity*, *n* (%)		
Afro-Caribbean	47 (6.0)	6 (3.5)
Caucasian	623 (80.0)	164 (95.9)
Melanesian Islands	109 (14.0)	–
Missing	–	*1 (0.6)*
*Comorbidities*, *n* (%)		
Tobacco use	62 (8.4)	6 (3.5)
High blood pressure	2 (0.3)	0 (0.0)
Diabetes	0 (0.0)	1 (0.6)
Asthma	47 (6.1)	23 (13.5)
No comorbidity	675 (86.7)	142 (83.0)
*Acute SARS-CoV-2 infection symptoms*, *n* (%)		
At least one symptom^1^	181 (23.7)	101 (59.1)
1 symptom	90 (11.8)	14 (8.2)
2 or 3 symptoms	59 (7.7)	36 (21.0)
> 3 symptoms	32 (4.2)	51 (29.9)
No symptoms	580 (76.3)	70 (40.9)
Specific symptom: Ageusia/Anosmia	24 (3.2)	22 (12.9)
COVID(+) at inclusion (COVID(+)_prevalent_)^2^, *n* (%)	52 (6.7)	27 (15.8)
Known positive RT-PCR within 3 months prior to inclusion	15 (28.8)	17 (63.0)
Positive serology	49 (94.2)	27 (100.0)
COVID(+) during follow-up (COVID(+)_incident_), *n* (%)	206 (28.3)	NA
Positive RT-PCR	192 (93.2)	NA
Positive serology without previous positive PCR		NA
Among those with positive RT-PCR:	6.8)	
Seroconversion (+ serology after + PCR)	155 (80.7)	NA
No seroconversion^3^	25 (13.0)	NA

SD: Standard deviation; BMI: body mass index

^1^Including anosmia/ageusia, from Day 0 questionnaire

^2^Prevalent cases, previous positive SARS-COV-2 RT-PCR or serology

^3^Only 180/192 + PCR players with available samples for serology testing after + PCR

^4^The student population included representation from 27 sporting disciplines, predominant among which were rugby (16%), handball (13%), judo (11%), soccer (7%) and athletics (7%)

### Biomarkers

711 athletes underwent hematological and biochemical analysis in the month prior to inclusion, with no identified abnormalities. CRP, troponin I and D-Dimer levels are included in Table 2. For COVID(+)_incident_ players, biomarkers were available pre- and post-SARS-CoV2 infection. The proportion of athletes with abnormal values was comparable pre- and post-infection [pre- vs post-COVID(+)_incident_; CRP 5.6% vs 8.7%; *P* = 0.40; troponin I; 5.9% vs 5.9%; *P* = 0.99]. When comparing pre- and post-infection sample pairs, D-Dimer levels (median (IQR) pg/ml); 231 (215; 270) vs 270 (215; 318), *P* = 2.10^–4^) as well as proportion of athletes with values above the upper limit threshold (7.9% vs 17.3%, *P* = 0.01) increased.

**Table 2:** Biological markers according to COVID status

Biomarkers^1^	F-NLR Rugby players, *n* = 779	Student athletes, *n* = 171
**CRP**, mg/L		
COVID(−)*Athletes*, median (Q1; Q3), D0	1.1 (0.9; 4.0)	0.6 (0.3; 1.5)
* Athletes with levels* > *5 mg/L, n %*	47 (9.0)	9 (6.3)
Among COVID(+)_incident_ *Athletes* with paired pre-and post-infection samples, *n* = 161:		
Pre-COVID time point, median (Q1; Q3)	1.5 (1.0; 4.0)	NA
* Athletes with levels* > *5 mg/L, n %*	9 (5.6)	NA
Post-COVID time point median (Q1; Q3)	2.0 (1.0; 4.0)	NA
* Athletes with levels* > *5 mg/L, n %*	14 (8.7)	NA
**Troponin,** ng/L		
COVID (−)*Athletes*, median (Q1; Q3), D0	5.7 (3.8; 10.0)	NA
* Athletes with levels* > *34 ng/L, n %*	11 (2.1)	NA
Among COVID(+)_incident_ *Athletes* with paired pre-and post-infection samples, *n* = 136:		
Pre-COVID time point, median (Q1; Q3)	5.1 (4.1; 10.5)	NA
* Athletes with levels* > *34 ng/L, n %*	8 (5.9)	NA
Post-COVID time point, median (Q1; Q3)	5.1 (4.7; 13.0)	NA
* Athletes with levels* > *34 ng/L, n %*	8 (5.9)	NA
**D-Dimer**, ng/mL		
COVID(−)*Athletes*, median (Q1; Q3), D0	270.0 (215.0; 285.0)	NA
* Athletes with levels* > *400 ng/ml, n %*	43 (8.3)	NA
Among COVID(+)_incident_ *Athletes* with paired pre-and post-infection samples, *n* = 139		
Pre-COVID time point, median (Q1; Q3)	231.0 (215.0; 270.0)	NA
* Athletes with levels* > *400 ng/ml, n %*	11 (7.9)	NA
Post-COVID(+) time point, median (Q1; Q3)	270.0 (215.0; 318.0)	NA
* Athletes with levels* > *400 ng/ml, n %*	24 (17.3)	NA

^1^Threshold for normal values was < 5 mg/L; < 34 ng/ml, and < 400 ng/mL for CRP, troponin I and D-Dimers, respectively. COVID(−) athletes: inclusion (D0) values described for those never having any evidence of prevalent or incident infection during the study (i.e., excluding also pre-infection values of later COVID(+)_incident_). For the COVID(+)_incident_ players subgroup with laboratory assessments available pre- and post-SARS-CoV2 infection, median (Q1; Q3) days after infection was 7 (7; 12)

### Cardiac investigations

Overall results of cardiovascular testing are summarized in Fig. 3 and Table 3. Of note, athletes with one abnormality in either ECG, stress test, echocardiography or CMR had no abnormalities in the other tests. Characteristics of athletes with abnormal cardiac investigations are included in Table 4.

**Table 3:** Cardiac assessment according to COVID status

Cardiac assessment	F-NLR Rugby players, *n* = 779	Student Athlete, *n* = 171
**ECG Abnormalities**, *n* (%)		
COVID (−) athletes, *n* (%)	2 (0.3)	0 (0.0)
Post-COVID(+), new abnormalities^1^	6 (2.9)	0 (0.0)
**Echocardiography abnormalities**, **n** (%)		
Post-COVID(+)^2^	1 (0.5)	0 (0.0)
**Stress test abnormalities**, **n** (%)		
Post-COVID(+)^3^	7 (3.4)	3 (12.0)
**CMR abnormalities**^4^		
COVID(−), *n* (%)		
Non-specific	2 (7.1)	NA
Specific	1 (3.6)	NA
Post-COVID(+), *n* (%)		
Non-specific	10 (11.1)	2 (16.7)
Specific	0 (0.0)	1 (8.3)

^1^All F-NRL athletes underwent ECG at inclusion. The ECG was compared to an ECG performed during the previous year. For COVID(+)_incident_ F-NRL players, the post-COVID ECG was compared to pre-COVID ECG. All sport students of the University of Bordeaux underwent an inclusion ECG

^2^COVID(+)_prevalent_ and COVID(+)_incident_ athletes underwent echocardiography at the end of the isolation period. Any COVID(+) prevalent student athletes (history of positive SARS-CoV-2 PCR in the previous 6 months and/or positive serology at inclusion) underwent echocardiography

^3^All COVID(+) athletes underwent stress testing. ^4^CMR was performed in 130 athletes [102 COVID(+) and 28 COVID(−)]

**Table 4:** Participants with at least one cardiologic abnormality on ECG/echocardiography/MRI/stress test according to COVID status

Study Group	Ethnic Group	Abnormal resting ECG	Abnormal stress test	Abnormal Echocardiogram	Fibrosis on CMR	Clinical events	COVID (+)	Abnormal Troponin (> 34)	Abnormal D-Dimer (> 400)
F-NRL player	Caucasian	–	Yes	–	–	–	Yes	–	–
F-NRL player	Caucasian	–	–	–	Non–specific	–	Yes	–	–
F-NRL player	Caucasian	–	–	–	Non–specific	–	Yes	–	–
F-NRL player	Caucasian	Yes	–	–	–	–	Yes	–	–
F-NRL player	Caucasian	–	Yes	–	–	–	Yes	–	–
F-NRL player	Caucasian	–	Missing	Missing	Yes	–	No	Yes	–
F-NRL player	Caucasian	–	–	–	Non–specific	–	Yes	–	–
F-NRL player	Caucasian	–	–	–	Non–specific	–	Yes	–	–
F-NRL player	Caucasian	–	–	Yes	–	–	Yes	–	Yes
F-NRL player	Caucasian	–	–	–	Non–specific	–	Yes	–	Yes
F-NRL player	Ilian-Melanesian	–	Yes	–	Missing	–	Yes	–	Yes
F-NRL player	Caucasian	–	–	–	Non–specific	–	Yes	Yes	–
F-NRL player	Ilian-Melanesian	–	Yes	–	Missing	–	Yes	Yes	Yes
F-NRL player	Ilian-Melanesian	–	Yes	–	Missing	–	Yes	–	Yes
F-NRL player	Afro-Caribbean	Yes	–	–	Missing	–	Yes	–	–
F-NRL player	Caucasian	–	–	–	Non–specific	–	No	–	–
F-NRL player	Caucasian	–	–	–	–	Ventricular Tachycardia	Yes	Missing	Missing
F-NRL player	Caucasian	–	–	–	Non–specific	–	Yes	–	–
F-NRL player	Caucasian	–	Yes	–	–	–	Yes	–	–
F-NRL player	Caucasian	–	–	–	Non–specific	–	Yes	–	–
F-NRL player	Caucasian	Yes	–	–	Missing	–	Yes	–	–
F-NRL player	Caucasian	–	–	–	Non–specific	–	Yes	–	–
F-NRL player	Caucasian	–	Missing	Missing	Non–specific	–	No	–	–
F-NRL player	Caucasian	Yes	–	–	Missing	–	Yes	Missing	Missing
F-NRL player	Caucasian	Yes	Missing	Missing	Missing	–	No	Missing	–
F-NRL player	Caucasian	Yes	–	Missing	Missing	–	Yes	Missing	Missing
F-NRL player	Caucasian	Yes	–	–	Missing	–	No	Missing	Missing
F-NRL player	Caucasian	Yes	–	–	–	–	Yes	–	–
F-NRL player	Caucasian	–	–	–	Non–specific	–	Yes	–	–
F-NRL player	Caucasian	–	Yes	–	Missing	–	Yes	–	–
Student Athl	Caucasian	–	Yes	–	–	–	Yes	Missing	Missing
Student Athl	Caucasian	–	Yes	–	Missing	–	Yes	Missing	Missing
Student Athl	Caucasian	–	Yes	–	–	–	Yes	Missing	Missing
Student Athl	Caucasian	–	–	–	Yes	–	Yes	Missing	Missing
Student Athl	Caucasian	–	–	–	Non–specific	–	Yes	Missing	Missing
Student Athl	Caucasian	–	–	–	Non–specific	–	Yes	Missing	Missing

‘–’, Normal assay; Student Athl., Student athletes

#### 12-lead electrocardiogram

No baseline ECG abnormalities were identified in the student cohort [COVID(−) or COVID(+)]. Among COVID(−) F-NRL rugby players, 2 (0.3%) had new ECG abnormalities at inclusion, compared to an ECG performed during the previous year (inferior/lateral T wave inversion and a ventricular ectopic beat). Among COVID(+) F-NRL rugby players, a new ECG abnormality was detected in 6 (2.9%): 1 COVID(+)_prevalent_ subject (inverted T waves in septal leads), and 5 COVID(+)_incident_ subject (inverted T waves: septal leads [*n* = 2], inferior and lateral leads [*n* = 2], septal and lateral leads [*n* = 1]).

#### Echocardiography

One (0.5%) COVID(+) F-NRL rugby players presented with a new regional wall motion abnormality on echocardiography post-COVID infection. None of the 25 COVID(+)_prevalent_ students who underwent echocardiography had any abnormalities.

#### Stress test

During exercise stress testing, 7/206 (3%) COVID(+) F-NRL rugby players had isolated ventricular ectopy (at an average performance of 327.4 ± 55 Watts with maximum systolic BP values of 187.9 ± 25.7 mmHg and diastolic BP of 82.5 ± 15.3 mmHg). In the students’ group, 3/25 (12%) had exercise-induced isolated ventricular ectopy (at 222 ± 54 Watts, with max systolic BP values at 157 ± 20 mmHg and diastolic BP at 70 ± 7 mmHg).

#### Cardiac MR

MRI images were analyzed in 130 athletes (F-NRL players and students). In the COVID(+) group (*n* = 102, 62% asymptomatic, 38% mildly symptomatic), MRI was acquired 51 ± 37 days post-COVID-19 diagnosis. CMR findings are presented in Additional file 1: Table 1. As expected, these highly trained athletes showed ventricular volumes and ejection fraction consistent with exercise-induced remodeling. There were no regional wall motion abnormalities or pericardial abnormalities identified. Native T1 values were within the normal range. Edema was detected in 1 (4.3%) COVID(−) athletes and in none of the COVID(+) athletes.

**Fig. 4:**
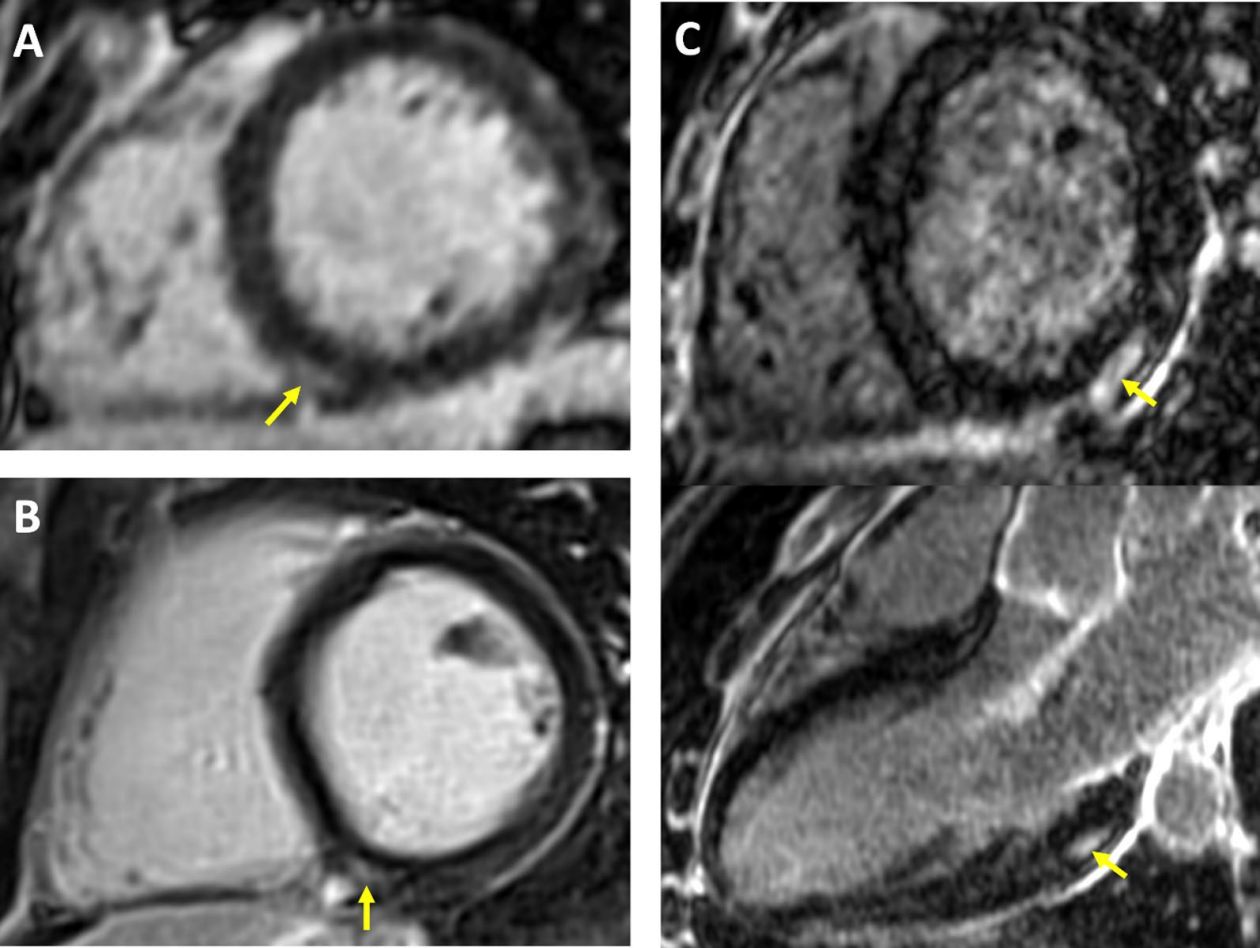
Late gadolinium enhancement CMR findings in athletes following mildly symptomatic COVID-19 infection. *Yellow* arrows indicate areas of LGE. A-B: small focal intramural LGE in the vicinity of the posterior RV insertion point. This pattern observed in equal proportions of COVID(+) and COVID(−) athletes, and considered as non-specific because suggestive of physiological remodeling. C: Intramural and sub-epicardial LGE lesion on infero-latero-basal LV segment, shown in short axis and 3-chamber views (top and bottom panel, respectively). The patient had experienced a mild symptomatic SARS-CoV-2 infection 65 days prior to the CMR study, with no clinical signs of myocarditis. Echocardiography, ECG and troponin tests were negative at inclusion. This CMR finding being suggestive of a post-inflammatory origin, scarring secondary to COVID-related myocarditis could not be ruled out

Small LGE lesions (median volume 3 mL) were detected in 16 (12%) athletes. Examples of LGE positive images in COVID(+) athletes are shown in Fig. 4. LGE was considered a non-specific finding related to physiological remodeling in 14 (11%), with a similar distribution between COVID(+) and COVID(−) populations [12% COVID(+); 8% COVID(−)]. LGE patterns indicative of pathological scarring were detected in 2 (1.6%) athletes, one from the COVID(−) group and one from the COVID(+) group. The F-NRL rugby player from the COVID(−) group was diagnosed with possible distal vasculitis based on the finding of multiple micro-infarcts and edema on MRI, with an associated subacute troponin I rise. The student COVID(+) athlete with scarring on LGE imaging had experienced mild COVID symptoms (only anosmia) 65 days prior to the MRI with no clinical signs of myocarditis, and no persisting symptoms at the time of the MRI study. Echocardiography and ECG were normal in this subject at the time of inclusion. The post-SARS-CoV2 infection MRI showed sub-epicardial scar on the infero-latero-basal left ventricle with no edema. Because this scar distribution is consistent with a post-inflammatory process, a potential diagnosis of scarring secondary to COVID-related myocarditis was considered.

### Subgroup analysis

There were no differences in the proportion of players presenting with a biomarker or cardiac abnormalities based on ethnic groups (32/787 (4.1%) Caucasians; 3/109 (2.7%) Melanesian; 1/53 (1.9%) Afro-Caribbean). The proportion of athletes with biomarker or cardiac abnormalities did not vary according to BMI. (BMI < 30 [*n* = 635] versus > 30 [*n* = 315]; CRP > 5: 42 (7.1%) vs 25 (8.6%); troponin I > 34: 12 (2.8%) vs 10 (3.4%); D-Dimer > 400: 35 (8.0%) vs 26 (9.0%)); ECG abnormalities: 6 (1.0%) vs 2 (0.7%); stress test abnormalities: 6 (3.4%) vs 4 (4.2%); and echocardiographic abnormalities: 1 (0.6%) vs 0 (0.0%).

### Clinical outcomes

The only recorded adverse clinical event was ventricular tachycardia in a patient with a previous SARS-CoV-2 infection, which occurred during a match. The subject had completed a cardiology work-up 64 days prior to the event. Post-COVID tests did not demonstrate repolarization or rhythm abnormalities on the resting or exercise ECG. Echocardiography and CMR imaging in the acute phase did not demonstrate any abnormalities. All biomarkers were within normal limits. The player underwent ablation of a focal ventricular arrhythmia from the right ventricular infundibulum and was able to return to competitive sport.

## Discussion

The main findings of the present study, which included a large cohort of elite- and high-level athletes, were as follows: (1) The rate of adverse clinical cardiac events after mild/asymptomatic COVID-19 is very low, despite resumption of high-level training, (2) in the vast majority of athletes, biomarkers of cardiac injury remain in the normal range after a mild/asymptomatic COVID-19, (3) the incidence of structural cardiac abnormalities attributable to COVID-19 on echocardiography and/or CMR is very low, (4) the burden of arrhythmias is low among athletes with mild/asymptomatic COVID, and (5) among the small subset of athletes with structural/electrical abnormalities potentially related to mild COVID-19, the abnormalities are subtle and restricted to a single cardiac investigation.

In keeping with our findings, in a large observational cohort from 42 USA colleges/universities, Moulson also reported a low rate of clinical events and abnormalities on cardiac evaluation (on ECG, troponin, echocardiography and CMR in selected cases) [21]. Importantly, in the present study, in addition to university athletes, we included a large cohort of > 750 professional rugby athletes, who routinely undertake a significantly higher exercise volume and intensity. More recently, Martinez and colleagues reported a low incidence of cardiac abnormalities in a similarly sized cohort of professional athletes. Importantly, however, MRI was performed in a very small subset of their cohort, and comparative data from COVID negative control athletes was not available [22]. Furthermore, we systematically performed stress testing, which allowed quantification of functional capacity and arrhythmia monitoring on exercise. Our results consolidate and build upon the results from the aforementioned observational registries [11, 19–23] and demonstrate that despite professional-level intensity of exercise, mild/asymptomatic SARS-CoV-2 infections are associated with a very low of adverse cardiac effects.

Comprehensive cardiac evaluation with ECG, echocardiography, stress tests, and indeed a high-resolution protocol CMR, failed to identify any abnormalities in the vast majority of our cohort. The rarity of ECG abnormalities in our COVID(+) athletes raises the question as to whether systematic ECG screening in required. During stress testing, repolarization abnormalities were very rare. Only a minority of athletes had exercise-induced isolated ventricular ectopy. In keeping with previous reports, the identification of abnormalities following echocardiographic analysis was also extremely low [21, 24]. Overall, the favorable cardiovascular outcomes indicate that the aforementioned investigations are not routinely indicated for mild/asymptomatic COVID-19 and should be reserved for symptomatic athletes. In agreement with prior evidence [21, 24–27], we recommend a simple rest period of 7 to 10 days before the gradual resumption of physical activity. We would, however, recommend caution when extrapolating these findings to emerging SARS-CoV-2 variants.

In the present study, state-of-the-art CMR, including high-resolution LGE, yielded a low rate of abnormalities in asymptomatic/mildly symptomatic COVID-19(+) athletes. CMR-based diagnosis of acute myocarditis relies on the identification of myocardial abnormalities on T1 and T2 imaging [28]. Unfortunately, this strategy is not sensitive after the inflammatory phase, because once edema has resolved, the inflammatory origin remains a working hypothesis and the temporal relationship with the initial insult remains unknown. This is a critical in athletes because of a higher prevalence of CMR-detected sub-clinical myocardial fibrosis due to physiological remodeling [29]. Most prior CMR series in COVID(+) patients have focused on hospitalized patients with a troponin rise, reporting a spectrum of myocardial injuries secondary to ischemic and non-ischemic mechanisms [9]. Initial series in less symptomatic COVID(+) cohorts have also reported an unexpectedly high rate of subclinical myocarditis on CMR, including among athletes [17, 30]. Our results indicate that the prevalence of scarring is very low, which is in line with several recent studies [11, 19–21, 24]. The discrepancy with the earlier CMR series could be explained by selection bias, and by the inappropriate attribution of non-specific findings to COVID-19. Overall, our results suggest that in the context of SARS-CoV-2 infection, as with other viruses with known cardiac tropism, CMR indications should be limited to athletes with clinically suspected myocarditis, or to those with positive troponin and/or echocardiography.

Consistent with recent reports, troponin I and CRP levels were in the normal range in the majority of our cohort with mild/asymptomatic COVID-19 [17, 21, 31]. In the rugby F-NRL cohort, we observed a proportion of athletes with increased D-Dimer levels after COVID-19. Of note, the baseline values of these parameters have been reported to be significantly higher in some athletes compared to the general population [32–34]. Rugby players are regularly exposed to sport-related trauma, which could potentially contribute to even higher D-Dimer levels. Caution is therefore needed when interpreting these biomarkers in rugby players the context of COVID-19, with or without associated myocardial lesions.

We did not find differences in the proportion of cardiac abnormalities according to ethnicity, in particular in our relatively large cohort of Ilian-Melanesian descent, who have rarely been studied in the literature [35]. In contrast to recently published work reporting a linear correlation between BMI and severity of COVID-19 infections in a young population [36], we did not demonstrate differences in the prevalence of biomarker/cardiac abnormalities between athletes with BMI < 30 kg/m^2^ and those with > 30 kg/m^2^. Beyond the potential contribution of intense physical activity on enhancing immunity [37], this finding argues for considering the fact that, at least in sportsmen, the fat mass alone rather than the overall body mass as a risk factor for developing severe COVID-19 infections, and in particular, cardiac sequelae of COVID-19 infections.

## Limitations

In contrast to the study of Brito et al. [30], we did not measure left ventricular longitudinal strain during echocardiographic analysis. Based on previous reports indicating lower baseline longitudinal strain in some athletes [38], this parameter was not deemed optimal for functional assessment in our cohort. Given the clinical context, increased D-Dimer levels were not systematically investigated with pulmonary angiography or pulmonary scintigraphy to eliminate potential differential diagnoses of embolic complications. However, the presence of elevated D-Dimer levels at inclusion in COVID(−) athletes, the excellent outcome in COVID(+) athletes exposed to a whole competitive season and the absence of a subsequent diagnosis of pulmonary embolism suggest that the elevated D-Dimer is likely to be attributable repeated hemorrhagic muscular micro- and macro-trauma related to intense exercise. Finally, there were significant differences in the protocols for investigation of F-NRL and student athletes and the two cohorts were not systematically matched in terms of baseline characteristics. In particular, we anticipated the fact that the population of F-NRL players are exclusively male and play exclusively rugby by including the student athletes population, 50% of whom are female and who play a variety of 27 sports. The outcomes in the mixed student population were not significantly different from the exclusively male F-NRL population. There were also no differences when comparing to a strictly female population of student athletes.

## Conclusions

In population of high-level athletes, pauci or asymptomatic COVID-19 was not associated with an increased risk of cardiac events. Electrical, morphological and functional assessment of the heart was consistently normal, even when using high-resolution CMR, indicating an absence of significant cardiac impact. Consequently, in keeping with recent recommendations [39, 40], systematic cardiac investigation prior to resumption of intense amateur or indeed professional sport could be significantly reduced, or even eliminated.

## Supplementary Information

**Additional file 1.** **Supplementary Table:** MRI findings.

## Data Availability

The datasets used and/or analyzed during the current study are available from the corresponding author on reasonable request.

## Abbreviations

COVID-19: Coronavirus disease2019
ECG: Electrocardiogram
VPB: Ventricular premature beat
CMR: Cardiac magnetic resonance
SARS-CoV-2: Severe acute respiratory syndrome coronavirus 2
F-NRL: French National Rugby League
METS: Metabolic equivalents of tasks.
SD: Standard deviation
LGE: Late gadolinium enhancement

## References

[1] Guo T, Fan Y, Chen M (2020). Cardiovascular implications of fatal outcomes of patients with coronavirus disease 2019 (COVID-19). JAMA Cardiol.

[2] Shi S, Qin M, Shen B (2020). Association of cardiac injury with mortality in hospitalized patients with COVID-19 in Wuhan. China JAMA Cardiol.

[3] Wang D, Hu B, Hu C (2020). Clinical characteristics of 138 hospitalized patients with 2019 novel coronavirus-infected pneumonia in Wuhan. China JAMA.

[4] Liu PP, Blet A, Smyth D, Li H (2020). The science underlying COVID-19: implications for the cardiovascular system. Circulation.

[5] Clerkin KJ, Fried JA, Raikhelkar J (2020). Coronavirus disease 2019 (COVID-19) and cardiovascular disease. Circulation.

[6] Basso C, Leone O, Rizzo S (2020). Pathological features of COVID-19-associated myocardial injury: a multicentre cardiovascular pathology study. Eur Heart J.

[7] Esposito A, Palmisano A, Natale L (2020). Cardiac magnetic resonance characterization of myocarditis-like acute cardiac syndrome in COVID-19. J Am Coll Cardiol Img.

[8] Ojha V, Verma M, Pandey NN (2021). Cardiac magnetic resonance imaging in coronavirus disease 2019 (COVID-19): a systematic review of cardiac magnetic resonance imaging findings in 199 patients. J Thorac Imaging.

[9] Kotecha T, Knight DS, Razvi Y (2021). Patterns of myocardial injury in recovered troponin-positive COVID-19 patients assessed by cardiovascular magnetic resonance. Eur Heart J.

[10] Puntmann VO, Carerj ML, Wieters I (2020). Outcomes of cardiovascular magnetic resonance imaging in patients recently recovered from coronavirus disease 2019 (COVID-19). JAMA cardiol.

[11] Joy G, Artico G, Kurdi H (2021). Prospective case-control study of cardiovascular abnormalities 6 months following mild COVID-19 in healthcare workers. JACC Cardiovasc Imaging.

[12] Finocchiaro G, Papadakis M, Robertus JL (2016). Etiology of sudden death in sports: insights from a United Kingdom regional registry. J Am Coll Cardiol.

[13] Peterson DF, Kucera K, Thomas LC (2020). Aetiology and incidence of sudden cardiac arrest and death in young competitive athletes in the USA: a 4-year prospective study. Br J Sports Med.

[14] Harmon KG, Asif IM, Maleszewski JJ (2015). Incidence, cause, and comparative frequency of sudden cardiac death in national collegiate athletic association athletes: a decade in review. Circulation.

[15] Bohm P, Scharhag J, Egger F (2021). Sports-related sudden cardiac arrest in Germany. Can J Cardiol.

[16] Wesslen L, Pahlson C, Lindquistt O (1996). An increase in sudden unexpected cardiac deaths among young Swedish orienteers during 1979–1992. Eur Heart J.

[17] Rajpal S, Tong MS, Borchers J (2021). Cardiovascular magnetic resonance findings in competitive athletes recovering from COVID-19 infection. JAMA Cardiol.

[18] Vago H, Szabo L, Dohy Z, Merkely B (2020). Cardiac magnetic resonance findings in patients recovered from COVID-19: initial experiences in elite athletes. JACC Cardiovasc Imaging.

[19] Daniels CJ, Rajpal S, Greenshields JT (2021). Prevalence of clinical and subclinical myocarditis in competitive athletes with recent SARS-CoV-2 infection. Results from the Big Ten COVID-19 cardiac registry. JAMA Cardiol..

[20] Starekova J, Bluemke DA, Bradham WS (2021). Evaluation for myocarditis in competitive student athletes recovering from coronavirus disease 2019 with cardiac magnetic resonance imaging. JAMA Cardiol.

[21] Moulson N, Petek BJ, Drezner JA (2021). SARS-CoV-2 Cardiac involvement in young competitive athletes. Circulation.

[22] Martinez MW, Tucker AM, Bloom OJ (2021). Prevalence of inflammatory heart disease among professional athletes with prior COVID-19 infection who received systematic return-to-play cardiac screening. JAMA Cardiol.

[23] Patel P, Thompson PD (2022). Diagnosing COVID-19 myocarditis in athletes using cMRI. Trends Cardiovasc Med.

[24] Löllgen H, Bachl N, Papadopoulou T (2021). Infographic. Clinical recommendations for return to play during the COVID-19 pandemic. Br J Sports Med.

[25] Kim JH, Levine BD, Phelan D (2021). Coronavirus disease 2019 and the athletic heart. Emerging perspectives on pathology, risks, and return to play. JAMA Cardiol..

[26] Kim JH (2021). Screening athletes for myocarditis with cardiac magnetic resonance imaging after COVID-19 infection—lessons from an english philosopher. JAMA Cardiol.

[27] Stokes KA, Jones B, Bennett M (2020). Returning to play after prolonged training restrictions in professional collision sports. Int J Sports Med.

[28] Ferreira VM, Schulz-Menger J, Holmvang G (2018). Cardiovascular magnetic resonance in nonischemic myocardial inflammation: expert recommendations. J Am Coll Cardiol.

[29] Malek LA, Bucciarelli-Ducci C (2020). Myocardial fibrosis athletes-Current perspective. Clin Cardiol.

[30] Brito D, Meester S, Yanamala N (2021). High prevalence of pericardial involvement in college student athletes recovering from COVID-19. JACC Cardiovasc Imaging.

[31] Clark DE, Parikh A, Dendy JM (2021). COVID-19 Myocardial pathology evaluated through screening cardiac magnetic resonance (COMPETE CMR). Circulation.

[32] Wilson MG, Hull JH, Rogers J (2020). Cardiorespiratory considerations for return-to-play in elite athletes after COVID-19 infection: a practical guide for sport and exercise medicine physicians. Br J Sports Med.

[33] Cantinotti M, Clerico A, Giordano R (2021). Cardiac Troponin-T release after sport and differences by age, sex, training type, volume, and intensity: a critical review. Clin J Sports Med.

[34] Sedaghat-Hamedani F, Kayvanpour E, Frankenstein L (2015). Biomarker changes after strenuous exercise can mimic pulmonary embolism and cardiac injury–a metaanalysis of 45 studies. Clin Chem.

[35] Johnson C, Forsythe L, Somauroo J (2018). Cardiac structure and function in elite native Hawaiian and Pacific islander Rugby Football League athletes: an exploratory study. Int J Cardiovasc Imaging.

[36] Hendren NS, de Lemos JA, Ayers C (2021). Association of body mass index and age with morbidity and mortality in patients hospitalized with COVID-19: Results from the American Heart Association COVID-19 cardiovascular disease registry. Circulation.

[37] Nieman DC, Wentz LM (2019). The compelling link between physical activity and the body’s defense system. J Sport Health Sci.

[38] Caselli S, Montesanti D, Autore C (2015). Patterns of left ventricular longitudinal strain and strain rate in Olympic athletes. J Am Soc Echocardiogr.

[39] https:/www.anssm.org/Content/pdf-files/COVID19/NCAA_COVID-18-AUG-2021.pdf

[40] McKinney J, Connely KA, Dorian P (2021). Reflections and recommandations from a Canadian working group. Can J Cardiol.

