# Supplementary material for: Resuming Training in High-Level Athletes After Mild COVID-19 Infection: A Multicenter Prospective Study (ASCCOVID-19)
Source: Sports Med Open. 2022 Jun 25;8:83. doi: 10.1186/s40798-022-00469-0 (PMC9233721; doi:10.1186/s40798-022-00469-0)
Supplement: Supplementary file 1 — Additional file 1. Supplementary Table: MRI findings. [file 40798_2022_469_MOESM1_ESM.docx]

**SUPPLEMENTARY FILE 1**

**MATERIAL AND METHODS**

**MRI PROTOCOL**

The MRI protocol comprised cine imaging, T2-weighted imaging and T2 mapping, T1 mapping, and late gadolinium-enhanced (LGE) imaging using both a conventional breath-held 2-dimensional sequence and a respiratory-navigated inversion recovery-prepared 3-dimensional sequence at higher spatial resolution (voxel size 2.5 x 1.25 x 1.25mm) (7). MRI studies were anonymized, centralized and adjudicated in a core lab at the Bordeaux University Hospital. Images were analysed using CVI42 (Circle Cardiovascular Imaging, Calgary, Canada) to measure left and right ventricular volumes, ejection fraction, global longitudinal strain, left ventricular wall thickness, regional wall motion and pericardial effusion. T2 mapping images were analyzed to detect focal edema, and to measure a mean T2 value on the LV wall (ms). T1 mapping images were analyzed to measure a mean native T1 value on the LV wall (ms). LGE images were interpreted to detect myocardial or pericardial injury. LGE lesions were categorized as sub-endocardial, intramural or sub-epicardial, the former suggesting an ischemic origin, while the two latter indicating a non-ischemic origin. Small areas of intramural septal LGE in the vicinity of RV insertion points were categorized as non-specific, as these indicate physiological remodeling rather than reparative scarring, particularly in athletes (8). The volume of LGE lesions was measured using the full width at half maximum technique (ml).

**RESULTS**

**SUPPLEMENTARY TABLE: MRI FINDINGS**

|  | **Total (n=133)** | **COVID - (n=28)** | **COVID + (n=105)** |
| --- | --- | --- | --- |
| LGE imaging |  |  |  |
| definite scar | 2/130 (2%) | 1/28 (4%) | 1/102 (1%) |
| non-specific fibrosis | 14/130 (11%) | 2/28 (7%) | 12/102 (12%) |
| Scar location |  |  |  |
| anterior | 0/2 (0%) | 0/1 (0%) | 0/1 (0%) |
| septal | 0/2 (0%) | 0/1 (0%) | 0/1 (0%) |
| inferior | 0/2 (0%) | 0/1 (0%) | 0/1 (0%) |
| lateral | 2/2 (100%) | 1/1 (100%) | 1/1 (100%) |
| Scar transmural distribution |  |  |  |
| subendocardial | 1/2 (50%) | 1/1 (100%) | 0/1 (0%) |
| intramural | 1/2 (50%) | 0/1 (0%) | 1/1 (100%) |
| subepicardial | 0/2 (0%) | 0/1 (0%) | 0/1 (0%) |
| transmural | 0/2 (0%) | 0/1 (0%) | 0/1 (0%) |
| Scar volume (mL) | 3.0 ± 3.6 | 0.4 | 5.5 |
| RVEDVi (mL/m2) | 109 ± 17 | 108 ± 19 | 109 ± 17 |
| RVESVi (mL/m2) | 54 ± 10 | 53 ± 13 | 54 ± 10 |
| RVEF (%) | 51 ± 5 | 51 ± 6 | 51 ± 5 |
| RV wall motion abnormalities | 0/133 (0%) | 0/28 (0%) | 0/105 (0%) |
| LVEDVi (mL/m2) | 108 ± 16 | 110 ± 20 | 107 ± 15 |
| LVESVi (mL/m2) | 46 ± 9 | 48 ± 11 | 46 ± 8 |
| LVEF (%) | 57 ± 5 | 56 ± 4 | 57 ± 5 |
| LV wall motion abnormalities | 0/133 (0%) | 0/28 (0%) | 0/105 (0%) |
| Myocardial native T1 (ms) | 1003 ± 29 | 997 ± 28 | 1004 ± 30 |
| Myocardial T2 (ms) | 46 ± 2 | 47 ± 4 | 46 ± 2 |
| Edema on T2 STIR imaging | 1/125 (1%) | 1/23 (4%) | 0/102 (0%) |

Note: LGE imaging available in 130/133 of the total population (28/28 COVID - and 102/105 COVID +); RV volumes and EF available in 123/133 of the total population (25/28 COVID - and 98/105 COVID +); LV volumes and EF available in 127/133 of the total population (26/28 COVID - and 101/105 COVID +); Native T1 values available in 120/133 of the total population (25/28 COVID - and 95/105 COVID +); T2 values available in 91/133 of the total population (19/28 COVID - and 72/105 COVID +); T2 STIR imaging available in 128/133 of the total population (23/28 COVID - and 102/105 COVID +); EDVi: end disatolic volume index; EF: ejection fraction; ESVi: end-systolic volume index; LGE: late gadolinium enhancement; LV: left ventricle; RV: right ventricle.
